# Supplementary material for: The MicroRNA Ame-Bantam-3p Controls Larval Pupal Development by Targeting the Multiple Epidermal Growth Factor-like Domains 8 Gene (megf8) in the Honeybee, Apis mellifera
Source: Int J Mol Sci. 2023 Mar 17;24(6):5726. doi: 10.3390/ijms24065726 (PMC10054489; doi:10.3390/ijms24065726)
Supplement: Supplementary file 1 [file ijms-24-05726-s001.zip › Figures.pdf]

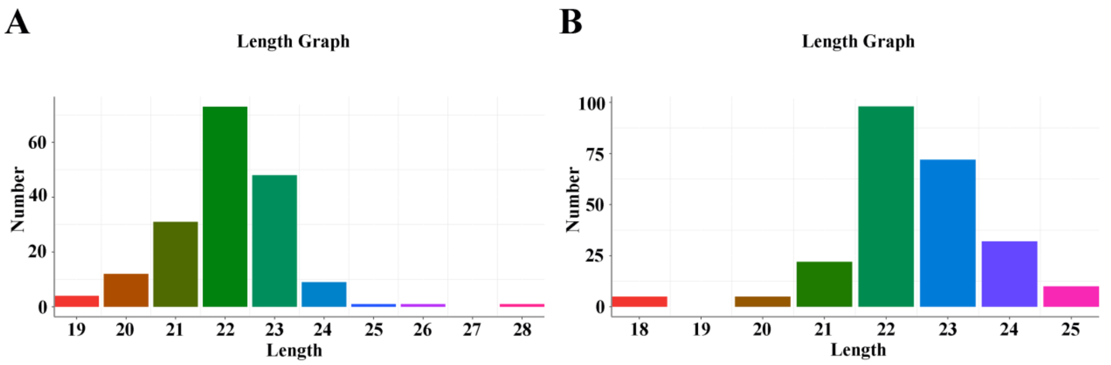

Figure S1 Length distribution of small RNAs. (A) Length distribution of known miRNAs sequences in different categories. (B) Length distribution of novel miRNAs sequences in different categories.

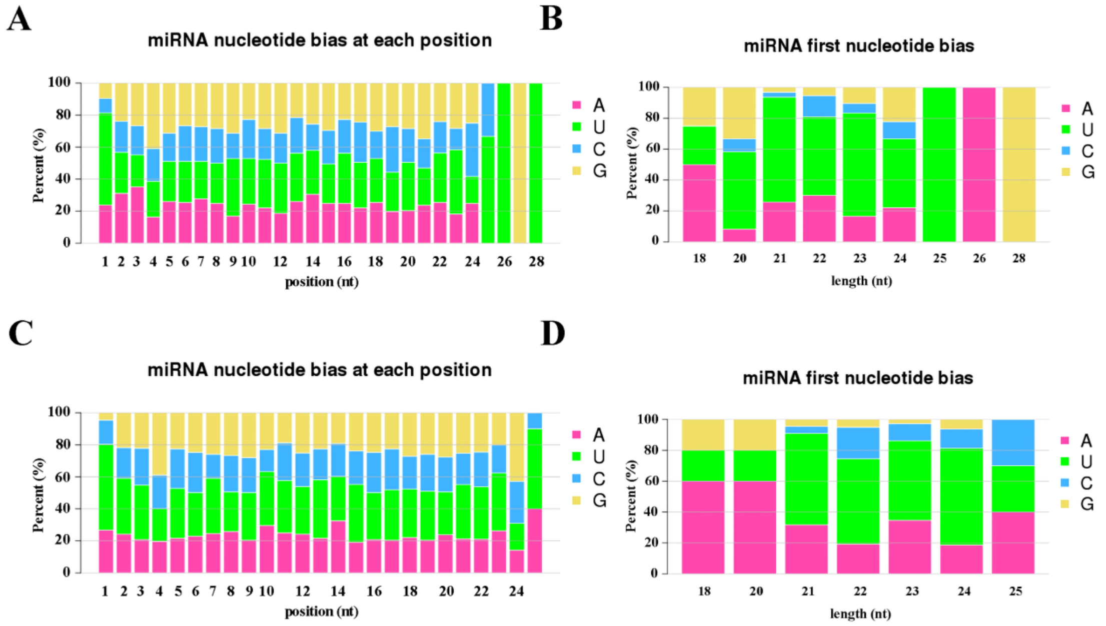

Figure S2 Nucleotide bias at each position (A), first-nucleotide bias (B) of known miRNAs. Nucleotide bias at each position (C), first-nucleotide bias (D) of novel miRNAs. Pw indicate the white eye pupa.

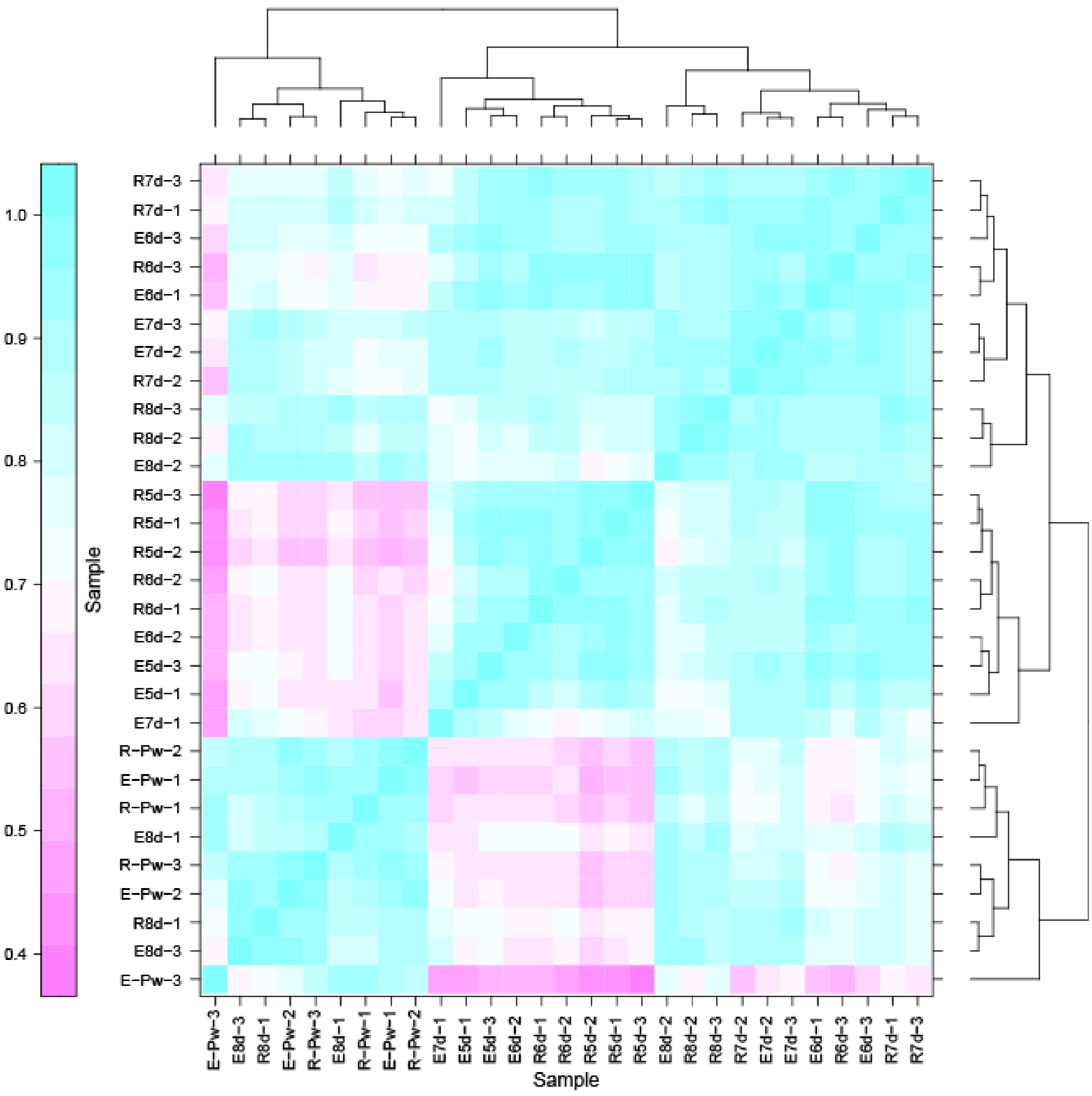

Figure S3 The Pearson’s correlation analysis of all honeybee larvae samples carried out in the present method. The feeding of 20E started with 2-day-old larvae and stoped when the larvae reach pupal weight (160 mg-180 mg) (E). Ringer solution was used as the control (R). Samples from 5-day-old larvae, 6-day-old (last-instar) larvae, 7-day-old larvae (prepupae), 8-day-old larvae (prepupae), and 10-day-old larvae (white-eye pupae, Pw) from different treatments were collected.

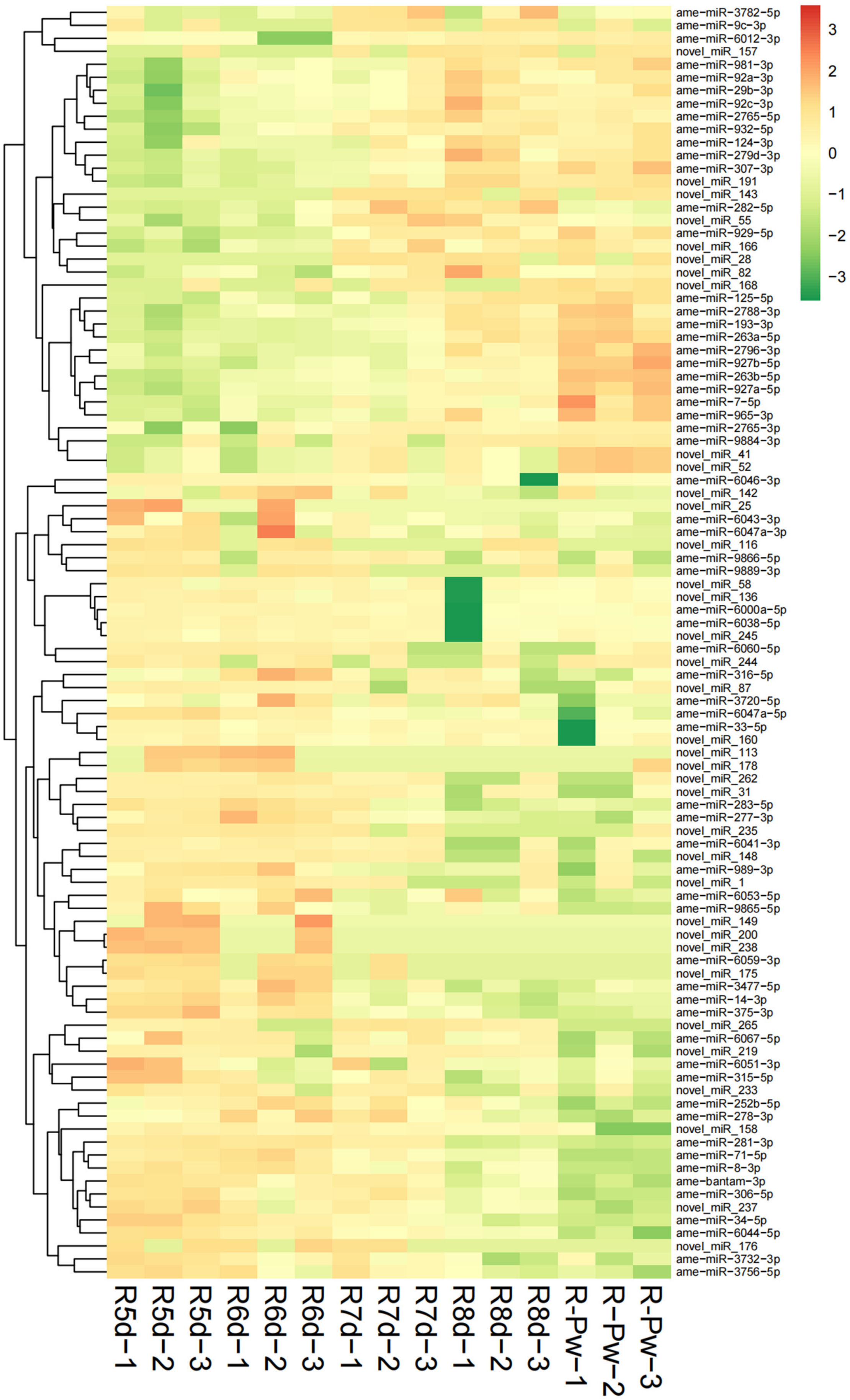
ame-miR-3782-5p
ame-miR-9c-3p
ame-miR-6012-3p
novel_miR_157
ame-miR-981-3p
ame-miR-92a-3p
ame-miR-29b-3p
ame-miR-92c-3p
ame-miR-2765-5p
ame-miR-932-5p
ame-miR-124-3p
ame-miR-279d-3p
ame-miR-307-3p
novel_miR_191
novel_miR_143
ame-miR-282-5p
novel_miR_55
ame-miR-929-5p
novel_miR_166
novel_miR_28
novel_miR_82
novel_miR_168
ame-miR-125-5p
ame-miR-2788-3p
ame-miR-193-3p
ame-miR-263a-5p
ame-miR-2796-3p
ame-miR-927b-5p
ame-miR-263b-5p
ame-miR-927a-5p
ame-miR-7-5p
ame-miR-965-3p
ame-miR-2765-3p
ame-miR-9884-3p
novel_miR_41
novel_miR_52
ame-miR-6046-3p
novel_miR_142
novel_miR_25
ame-miR-6043-3p
ame-miR-6047a-3p
novel_miR_116
ame-miR-9866-5p
ame-miR-9889-3p
novel_miR_58
novel_miR_136
ame-miR-6000a-5p
ame-miR-6038-5p
novel_miR_245
ame-miR-6060-5p
novel_miR_244
ame-miR-316-5p
novel_miR_87
ame-miR-3720-5p
ame-miR-6047a-5p
ame-miR-33-5p
novel_miR_160
novel_miR_113
novel_miR_178
novel_miR_262
novel_miR_31
ame-miR-283-5p
ame-miR-277-3p
novel_miR_235
ame-miR-6041-3p
novel_miR_148
ame-miR-989-3p
novel_miR_1
ame-miR-6053-5p
ame-miR-9865-5p
novel_miR_149
novel_miR_200
novel_miR_238
ame-miR-6059-3p
novel_miR_175
ame-miR-3477-5p
ame-miR-14-3p
ame-miR-375-3p
novel_miR_265
ame-miR-6067-5p
novel_miR_219
ame-miR-6051-3p
ame-miR-315-5p
novel_miR_233
ame-miR-252b-5p
ame-miR-278-3p
novel_miR_158
ame-miR-281-3p
ame-miR-71-5p
ame-miR-8-3p
ame-bantam-3p
ame-miR-306-5p
novel_miR_237
ame-miR-34-5p
ame-miR-6044-5p
novel_miR_176
ame-miR-3732-3p
ame-miR-3756-5p
R5d-1
R5d-2
R5d-3
R6d-1
R6d-2
R6d-3
R7d-1
R7d-2
R7d-3
R8d-1
R8d-2
R8d-3
R-Pw-1
R-Pw-2
R-Pw-3
3
2
1
0
-1
-2
-3

Figure S4 Hierarchical cluster analysis of all honeybee larvae samples. The green and red rectangles indicate downregulated and upregulated miRNAs, respectively. Samples from 5-day-old larvae, 6-day-old (last-instar) larvae, 7-day-old larvae (prepupae), 8-day-old larvae (prepupae), and 10-day-old larvae (white-eye pupae, Pw) from the control were collected.
