# Supplementary material for: The MicroRNA Ame-Bantam-3p Controls Larval Pupal Development by Targeting the Multiple Epidermal Growth Factor-like Domains 8 Gene (megf8) in the Honeybee, Apis mellifera
Source: Int J Mol Sci. 2023 Mar 17;24(6):5726. doi: 10.3390/ijms24065726 (PMC10054489; doi:10.3390/ijms24065726)
Supplement: Supplementary file 1 [file ijms-24-05726-s001.zip › Table S10.pdf]

Table S10 The miRNAs in each family

Family	miR_name
bantam	ame-bantam-3p
mir-467	novel_miR_149
mir-467	novel_miR_223
mir-467	novel_miR_90
mir-279	novel_miR_55
mir-279	ame-miR-279a-3p
mir-279	ame-miR-279d-3p
mir-279	ame-miR-996-3p
mir-2	ame-miR-13a-3p
mir-2	ame-miR-13b-3p
mir-2	ame-miR-2b-5p
mir-2	ame-miR-2-3p
mir-1277	novel_miR_72
mir-1277	novel_miR_35
mir-1277	novel_miR_16
mir-1277	novel_miR_214
mir-3747	novel_miR_69
mir-3747	ame-miR-3747a-5p
mir-3747	novel_miR_233
mir-3747	ame-miR-3747b-5p
mir-3747	novel_miR_242
mir-1677	novel_miR_36
mir-995	novel_miR_217
mir-276	ame-miR-276-3p
mir-1677	mir-1677
mir-995	novel_miR_217
mir-7	ame-miR-7-5p
mir-190	novel_miR_38
mir-190	ame-miR-190-5p
mir-14	ame-miR-14-3p
mir-14	novel_miR_22
mir-8	ame-miR-8-3p
mir-39	ame-miR-3791-3p
mir-39	novel_miR_54
mir-9	novel_miR_7
mir-9	ame-miR-9a-5p
mir-9	ame-miR-79-3p
mir-9	novel_miR_158
mir-9	ame-miR-9b-5p
mir-9	ame-miR-9c-3p
ame-miR-993-3p	mir-10
ame-miR-125-5p	mir-10

ame-miR-10-5p	mir-10
ame-miR-100-5p	mir-10
mir-263	ame-miR-263a-5p
mir-263	ame-miR-263b-5p
mir-219	ame-miR-219-5p
mir-iab-4	ame-miR-iab-4-5p
mir-278	ame-miR-278-3p
mir-277	ame-miR-277-3p
mir-252	ame-miR-252b-5p
mir-252	ame-miR-252a-5p
novel_miR_52	mir-629
mir-184	ame-miR-184-3p
mir-929	ame-miR-929-5p
mir-67	ame-miR-307-3p
ame-miR-965-3p	mir-965
ame-miR-965-5p	mir-965
novel_miR_234	mir-598
ame-miR-1-3p	mir-1
ame-miR-989-3p	mir-989
ame-miR-927a-5p	mir-927
ame-miR-6047b-3p	mir-1388
ame-miR-306-5p	mir-306
ame-miR-29b-3p	mir-29
ame-miR-6000a-3p	mir-675
ame-miR-927b-5p	mir-927
novel_miR_226	mir-135
ame-miR-283-5p	mir-216
ame-miR-137-3p	mir-137
ame-miR-981-3p	mir-981
ame-miR-2765-3p	mir-2765
novel_miR_203	mir-3543
novel_miR_52	mir-629
novel_miR_118	mir-1908
ame-let-7-5p	let-7
ame-miR-3718a-3p	mir-3718
ame-miR-282-5p	mir-282
ame-miR-12-5p	mir-12
ame-miR-3727-3p	mir-147
ame-miR-932-5p	mir-932
ame-miR-971-3p	mir-971
ame-miR-315-5p	mir-315
novel_miR_100	mir-3149
novel_miR_15	mir-1256
novel_miR_161	mir-1244

ame-miR-1000-5p	mir-1000
novel_miR_208	mir-15
ame-miR-9896-5p	mir-8069
novel_miR_87	mir-2944
novel_miR_201	mir-548
novel_miR_165	mir-1422
ame-miR-316-5p	mir-316
ame-miR-34-5p	mir-34
ame-miR-3770-5p	mir-7371
ame-miR-2796-3p	mir-2796
ame-miR-305-5p	mir-305
ame-miR-252a-5p	mir-252
novel_miR_119	mir-1271
ame-miR-318-3p	mir-3
novel_miR_138	mir-242_2
novel_miR_175	mir-9242
novel_miR_44	mir-2160
ame-miR-124-3p	mir-124
novel_miR_146	mir-4841
novel_miR_180	mir-968
ame-miR-6012-3p	mir-6012
ame-miR-275-3p	mir-275
ame-miR-193-3p	mir-193
ame-miR-31a-5p	mir-31
ame-miR-11-3p	mir-11
ame-miR-9892-3p	mir-9235
ame-miR-2944-3p	mir-2944
ame-miR-317-3p	mir-317
ame-miR-3477-5p	mir-3477
ame-miR-133-3p	mir-133
ame-miR-33-5p	mir-33
novel_miR_205	mir-2069
ame-miR-2765-5p	mir-2765
ame-miR-281-3p	mir-46
ame-miR-71-5p	mir-71
ame-miR-6067-5p	mir-2284
ame-miR-3478-3p	mir-3478
novel_miR_53	mir-281
