# Supplementary material for: The MicroRNA Ame-Bantam-3p Controls Larval Pupal Development by Targeting the Multiple Epidermal Growth Factor-like Domains 8 Gene (megf8) in the Honeybee, Apis mellifera
Source: Int J Mol Sci. 2023 Mar 17;24(6):5726. doi: 10.3390/ijms24065726 (PMC10054489; doi:10.3390/ijms24065726)
Supplement: Supplementary file 1 [file ijms-24-05726-s001.zip › Table S5..pdf]

Table S5 Base distribution frequency of known miRNAs

Position	A (Number)	U (Number)	G (Number)	C (Number)	U+G (Number)	A+C (Number)
1	42	101	16	17	118	58
2	55	45	34	42	87	89
3	62	35	32	47	82	94
4	29	39	36	72	111	65
5	46	44	31	55	99	77
6	45	45	39	47	92	84
7	49	41	38	48	89	87
8	44	44	38	50	94	82
9	30	63	28	55	118	58
10	43	50	43	40	90	86
11	39	53	34	50	103	73
12	33	55	33	55	110	66
13	46	53	39	38	91	85
14	54	48	29	45	93	83
15	44	43	37	52	95	81
16	44	55	37	40	95	81
17	39	50	44	43	93	83
18	45	48	30	53	101	75
19	35	43	50	48	91	85
20	36	53	37	50	103	73
21	39	38	30	57	95	69
22	34	41	26	32	73	60
23	11	24	8	17	41	19
24	3	2	4	3	5	7
25	0	2	1	0	2	1
26	0	2	0	0	2	0
27	0	0	0	1	1	0
28	0	1	0	0	1	0
