# Supplementary material for: The MicroRNA Ame-Bantam-3p Controls Larval Pupal Development by Targeting the Multiple Epidermal Growth Factor-like Domains 8 Gene (megf8) in the Honeybee, Apis mellifera
Source: Int J Mol Sci. 2023 Mar 17;24(6):5726. doi: 10.3390/ijms24065726 (PMC10054489; doi:10.3390/ijms24065726)
Supplement: Supplementary file 1 [file ijms-24-05726-s001.zip › Table S6.pdf]

Table S6 Base distribution frequency of novel miRNAs

Position	A (Number)	U (Number)	G (Number)	C (Number)	U+G (Number)	A+C (Number)
1	64	128	36	11	139	100
2	58	83	46	52	135	104
3	50	81	55	53	134	105
4	47	49	50	93	142	97
5	52	74	59	54	128	111
6	55	65	60	59	124	115
7	59	82	36	62	144	95
8	62	59	54	64	123	116
9	49	71	52	67	138	101
10	71	80	33	55	135	104
11	60	78	56	45	123	116
12	58	71	50	60	131	108
13	52	87	46	54	141	98
14	78	66	49	46	112	127
15	46	86	50	57	143	96
16	50	70	60	59	129	110
17	49	75	61	54	129	110
18	53	72	49	65	137	102
19	49	73	55	62	135	104
20	57	64	52	66	130	109
21	50	79	46	59	138	96
22	45	69	46	52	121	91
23	30	41	20	23	64	50
24	6	7	11	18	25	17
25	4	5	1	0	5	5
26	64	128	36	11	139	100
27	58	83	46	52	135	104
28	50	81	55	53	134	105
