# Supplementary material for: The MicroRNA Ame-Bantam-3p Controls Larval Pupal Development by Targeting the Multiple Epidermal Growth Factor-like Domains 8 Gene (megf8) in the Honeybee, Apis mellifera
Source: Int J Mol Sci. 2023 Mar 17;24(6):5726. doi: 10.3390/ijms24065726 (PMC10054489; doi:10.3390/ijms24065726)
Supplement: Supplementary file 1 [file ijms-24-05726-s001.zip › Table S7.pdf]

**Table S7 Summary of predicted miRNAs frome all samples**

| Sample ID | Number of known_miRNAs | Number of novel_miRNAs | Number of total miRNAs | miRNA with target | Target gene |
|---|---|---|---|---|---|
| R5d-1 | 150 | 110 | 260 | 161 | 1822 |
| R5d-2 | 149 | 137 | 286 | 178 | 1898 |
| R5d-3 | 153 | 144 | 297 | 193 | 2100 |
| R6d-1 | 137 | 82 | 219 | 125 | 1416 |
| R6d-2 | 145 | 82 | 227 | 144 | 1614 |
| R6d-3 | 143 | 76 | 219 | 131 | 1429 |
| R7d-1 | 152 | 112 | 264 | 153 | 1667 |
| R7d-2 | 153 | 109 | 262 | 163 | 1867 |
| R7d-3 | 147 | 90 | 237 | 138 | 1459 |
| R8d-1 | 132 | 45 | 177 | 109 | 1141 |
| R8d-2 | 148 | 95 | 243 | 142 | 1673 |
| R8d-3 | 150 | 103 | 253 | 144 | 1493 |
| R-Pw-1 | 129 | 39 | 168 | 96 | 1134 |
| R-Pw-2 | 154 | 111 | 265 | 160 | 1736 |
| R-Pw-3 | 152 | 96 | 248 | 150 | 1720 |
| E5d-1 | 154 | 142 | 296 | 178 | 1899 |
| E5d-3 | 163 | 166 | 329 | 203 | 2172 |
| E6d-1 | 144 | 98 | 242 | 141 | 1484 |
| E6d-2 | 139 | 81 | 220 | 127 | 1482 |
| E6d-3 | 147 | 106 | 253 | 148 | 1765 |
| E7d-1 | 161 | 153 | 314 | 194 | 2074 |
| E7d-2 | 164 | 138 | 302 | 186 | 1965 |
| E7d-3 | 155 | 123 | 278 | 166 | 1723 |
| E8d-1 | 136 | 63 | 199 | 118 | 1350 |
| E8d-2 | 139 | 58 | 197 | 123 | 1460 |
| E8d-3 | 146 | 80 | 226 | 137 | 1395 |
| E-Pw-1 | 148 | 75 | 223 | 133 | 1531 |
| E-Pw-2 | 140 | 64 | 204 | 120 | 1454 |
| E-Pw-3 | 139 | 78 | 217 | 126 | 1622 |
